# A comparative cross-sectional study on the quality of life in Grave’s disease patients: urban vs. rural perspectives

**DOI:** 10.3389/fpubh.2024.1345803

**Published:** 2024-08-21

**Authors:** Shivang Mishra, Anurag Kumar Singh, Sumit Rajotiya, Sourav Debnath, Sachin Kumar, Pratima Singh, Snehpreet, Preeti Raj, Mahaveer Singh, Hemant Bareth, Deepak Nathiya, Balvir Singh Tomar

**Affiliations:** ^1^Department of Pharmacy Practice, Nims Institute of Pharmacy, Nims University Rajasthan, Jaipur, India; ^2^Axel Pries Center for Biomedical Sciences and Public Health, Nims University Rajasthan, Jaipur, India; ^3^Global Center for Sustainable Development, Nims University Rajasthan, Jaipur, India; ^4^School of Public Health, University of Alberta, Edmonton, AB, Canada; ^5^Department of Pharmacology, National Institute of Medical Sciences, Nims University Rajasthan, Jaipur, India; ^6^School of Health Sciences, Faculty of Biology, Medicine and Health, The University of Manchester, Manchester Academic Health Science Center, Manchester, United Kingdom; ^7^Department of Endocrinology, National Institute of Medical Sciences, Nims University Rajasthan, Jaipur, India; ^8^Department of Clinical Studies, Fourth Hospital of Yulin (Xingyuan), Yulin, Shaanxi, China; ^9^Institute of Pediatric Gastroenterology and Hepatology, Nims University Rajasthan, Jaipur, India; ^10^Department of Clinical Sciences, Shenmu Hospital, Shenmu, Shaanxi, China

**Keywords:** Grave’s disease, HR-QoL, physical component summary score, mental component summary score, urban, rural

## Abstract

Grave’s disease affects numerous patients globally, but its impact on health-related quality of life (HR-QoL) in relation to geographical disparities remains under-explored. This cross-sectional study aimed to assess the influence of urban versus rural residence on HR-QoL among patients diagnosed with Graves’ Disease in Rajasthan, India. One hundred seven Graves’ disease patients from rural and urban endocrine centers were analyzed. The rural group included 52 patients (24 males, 28 females), averaging 38.9 ± 10.9 years of age, while the urban group had 55 (13 males, 42 females) with an average age of 39.1 ± 14.2 years. We found differences between rural and urban patients in terms of gender ratio, BMI, smoking habits, and obesity. Multivariable linear regression was used in both groups to determine the association between the baseline characteristics of Graves’ patients from both areas and HR-QOL. Health-related quality of life, assessed via the SF-36 questionnaire, indicated higher general health and role emotional scores among urban patients. Our study found that the duration of Graves’ disease in rural centers negatively impacted physical health scores. In urban patients, age and BMI influenced physical health, while gender and disease duration affected mental health scores in rural patients. Age impacted mental health in urban patients. Rural patients had a poorer quality of life compared to urban patients. Differences in gender distribution, BMI, smoking habits, and obesity rates revealed disparities in Graves’ disease between rural and urban patients in India, highlighting the need for better healthcare infrastructure and awareness in rural areas.

## 1 Introduction

Graves’ disease is an autoimmune thyroid disease, caused by the TSH receptor antibodies induced activation of the thyroid gland. Symptoms of the Graves’ disease range from distressing weight loss, palpitations, fatigue, heat intolerance to depression, and cognitive deficits. These wide-ranging symptoms significantly affect the patient’s quality of life, impacting their daily activities, occupational performance, emotional well-being, and social relationships, substantially diminishing their overall quality of life (1).

Most of the clinic-epidemiological studies on the Graves have focused on urban populations and ignored rural perspective (2, 3). In rural India, limited healthcare access, long travel times, less specialized care, and cultural or societal stigmas associated with chronic illnesses can significantly impact the trajectories and outcomes of the Graves’ disease in rural patients (4). The clinic-epidemiological data of Graves is skewed toward the urban population and does not represent the rural population with Graves.

This manuscript addresses this research gap by comparing the clinic-epidemiological spectrum of Graves’ disease in urban and rural settings. This study also investigates how geographical disparities can affect HR-QoL in patients with Grave’s disease.

## 2 Methods

### 2.1 Study design and population

The study is a cross-sectional study conducted at NIMS University’s two endocrine centers. The first endocrine center (urban center) is in the Raja Park of Jaipur, and the second endocrine center (rural) is located in the university hospital at the Chitanukalan village, Tehsil-Amber, Jaipur, Rajasthan, India (5). The study was conducted between March and June of 2023. To calculate the sample size for comparing the quality of life (QoL) in patients with Graves’ disease in rural and urban areas using the SF-36 questionnaire, we use the formula for comparing two means. Although there is no specific study on this topic, we extrapolate from past studies comparing QoL in rural and urban populations without Graves’ disease (Supplementary File 1) (6).

The inclusion criteria for this study were (a) >18 years of age, (b) confirmed diagnosis of Graves’ disease, and (c) willingness to provide informed consent for participation. Patients who did not meet the diagnostic criteria established by the European Thyroid Association or who showed evidence of other autoimmune diseases, such as type 1 diabetes, celiac disease, adrenal insufficiency, or hypogonadism, were excluded (7). Patients with coronary artery disease, chronic liver disease, chronic lung disease, and substance abuse disorder were also excluded from the study.

### 2.2 Study recruitment procedure

**Figure 1:**
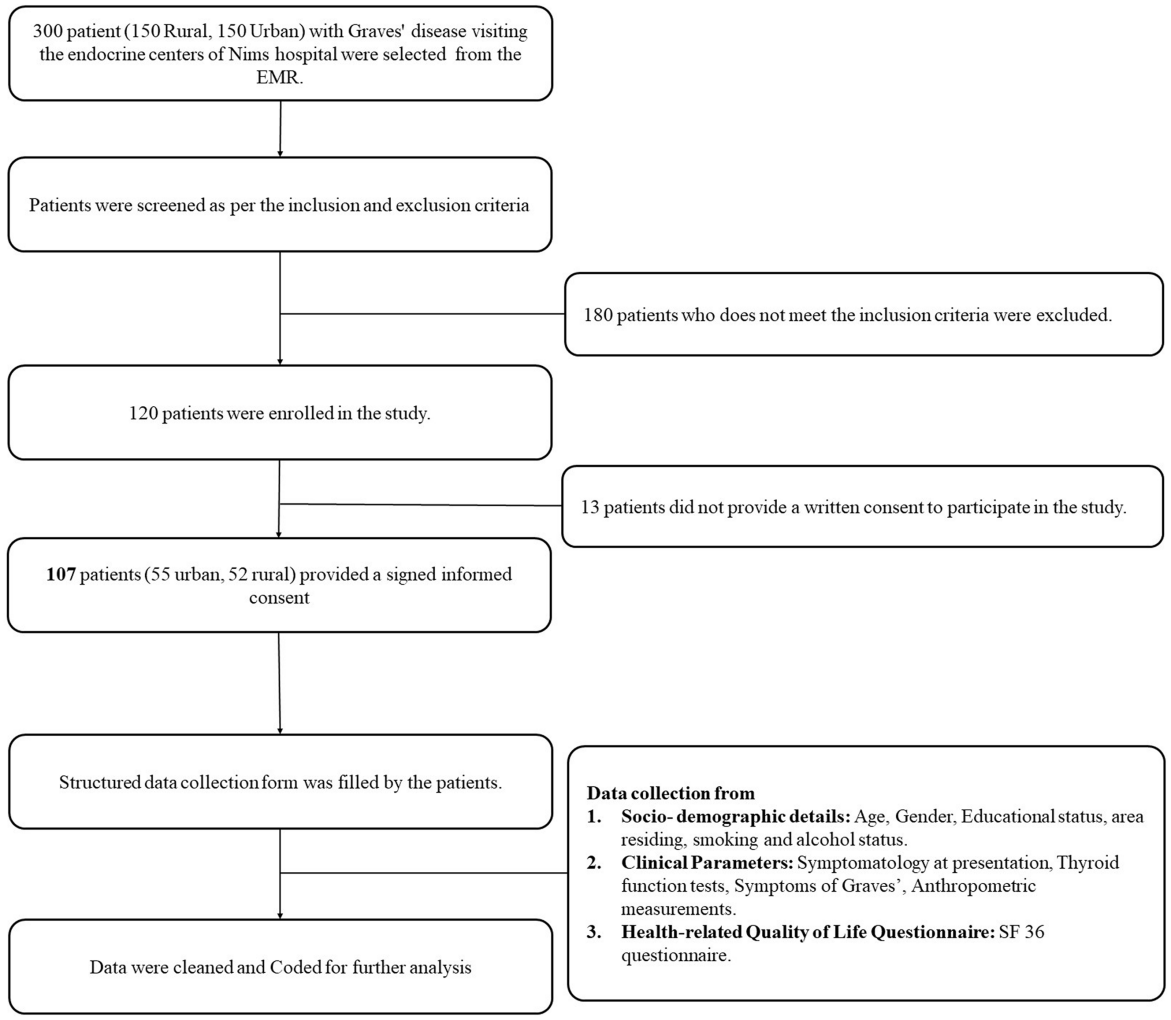
Flowchart illustrating the study methodology.

At NIMS University, the endocrinology department stores all patient-related information, including demographics, clinical history, examination, diagnosis, investigations, treatment, and follow-up, in the EMR (Healthplix). The EMR have a structured patient history and demonstration form. This form is used to record the clinical data. We selected 300 Graves’ disease patients from the EMR at both (urban and rural) centers. After screening for inclusion and exclusion criteria, we found 120 patients eligible for the study. Fifty-five patients from the urban endocrine center and 52 from the university hospital attached endocrine center consented to the study (Figure 1).

### 2.3 Collection of clinical data

We collected data from the EMR using specially designed forms and cross-checked it with in-person patient interviews. We gathered information about the patient’s age, gender, educational status, and residence (rural or urban). We also recorded detailed clinical history, including symptomatology at presentation (weight fluctuations, shortness of breath during exertion, palpitations, fatigue, anxiety, appetite irregularities, and moist or warm hands) and values of thyroid function tests (serum thyroid stimulating hormone, T4, and T3) at the time of diagnosis (8). Symptoms of Graves’ Orbitopathy, including eye pain (ophthalmalgia), swelling around the eyes (periorbital edema), and vision abnormalities (blurred vision, color desaturation, scotoma), were also recorded. We measured patients’ weight (in kilograms) and height (in meters) to calculate the body mass index (BMI) using the formula BMI = weight (kg)/height^2^ (meters squared). We used the World Health Organization’s classification of overweight (23.0–24.9 kg/m^2^) and obesity (BMI ≥ 25.0 kg/m^2^) (9). Patients’ smoking and alcohol status was categorized based on the National Center for Health Statistics guidelines by the Centers for Disease Control and Prevention (10, 11).

### 2.4 Health-related quality of life questionnaire

The patient’s health-related quality of life was assessed using the HRQOL 36-item SF-36 questionnaire. This commonly used questionnaire evaluates patients’ mental and physical well-being. The SF-36 questionnaire comprises eight scales that generate two summary measures: physical and mental health. The physical health summary scale consists of four scales: physical functioning (10 items), role-physical (four items), bodily pain (two items), and general health (five items). Vitality (four items), social functioning (two items), role-emotional (three items), and mental health (five items) comprise the mental health measure of emotional role (12). Energy and exhaustion levels, social involvement, the influence of emotional disorders on daily roles, and mental health are assessed in these domains (13, 14). The scoring followed RAND Healthcare, RAND Corporation’s SF-36 scoring instructions. Domain ratings range from 0 to 100, with higher scores indicating greater health-related quality of life and poorer scores (15–18).

### 2.5 Lab investigations

We did serum thyroid stimulating hormone (0.5–5.0 mIU/L), total T3 (0.9–2.8 ng/mL), and total T4 (5.4–11.5 μg/dL) (ECLIA, Vitros 5600 Integrated system, CA, USA) (19–21). Hyperthyroid patients who did not have clinical evidence (orbitopathy, dermopathy) of Graves’ disease were subjected to the Tc99 thyroid scan (Millenium MG, GE, Chicago, IL, USA) for uptake studies. The standard normal uptake was kept between 0.2 and 2.0%. Patients with a Tc99 uptake of less than 0.04% were labeled as having reduced uptake and excluded from the study. Hyperthyroid patients with Tc99 uptake >0.2% were labeled as normal/increased uptake, suggesting Graves’ disease (7). In patients who did not want the thyroid scan or had contraindications for it, serum thyroid stimulating hormone receptor antibodies (>1.75 IU/L) [Cobas e 801, Basel, Switzerland] were done to confirm the diagnosis of Graves’ disease (22).

### 2.6 Ethics

The current study was carried out with the approval of the Institutional Review Board (approval number: NIMSUR/IEC/2023/672) by the ethical principles outlined in the Declaration of Helsinki. All patients provided informed consent before participating in this observational study.

### 2.7 Statistical analysis

The data was analyzed by IBM SPSS software, version 28.0, based in Chicago, IL, USA. Continuous variables were presented using the mean and standard deviation, and categorical variables were expressed in frequency and proportion. The t-test was used to compare quantitative variables, while the chi-square was used to compare categorical variables, Mann–Whitney U test was employed to compare the non-parametric data. The determinants of the physical and mental components score of the SF-36 were computed using multivariable linear regression among urban and rural patients.

## 3 Results

### 3.1 Study population

Of the 120 eligible patients, 13 declined to participate in the study. As a result, 107 patients with Grave’s disease were included in the study, with 52 (24 male/28 females) from the university endocrine center (rural) center and 55 (13 male/42 female) from the urban endocrine center. The average age of patients from rural and urban centers was 38.9 ± 10.9 years (range 21–62) and 39.1 ± 14.2 years (range 18–75 years), respectively. In the patients presented to the rural center, there were seven graduates; at the urban center, there were 48 graduates, and no patients were illiterate.

The BMI of patients who were presented from the rural center was 20.94 ± 3.63 kg/m^2^, whereas, in the urban center, it was 24.26 ± 4.90 kg/m^2^ (Table 1).

**Table 1:** Baseline demographical parameters of the Graves patients enrolled at the rural and urban endocrine centers of the NIMS university.

Demographical parameters	Rural (*n* = 52)	Urban (*n* = 55)	*p*-value*
**Age** (mean ± SD, years)	38.96 ± 10.88	39.25 ± 14.06	0.100
**Age of diagnosis** (mean ± SD, years)	37.84 ± 10.84	37.29 ± 13.90	0.398
**Gender** (*n*)			**0.014**
Male	24 (46.1%)	13 (23.6%)	
Female	28 (53.8%)	42 (76.3%)	
**Education** (*n*)			**<0.001**
Illiterate	11 (21.1%)	0	
Primary school	18 (34.6%)	1 (0.18%)	
High school	12 (23.07%)	0	
Intermediate	4 (0.76%)	6 (10.9%)	
Graduate or postgraduate	7 (13.46%)	48 (87.2%)	
**BMI**^**ǂ**^ **(kg/m**^**2**^**)**	20.94 ± 3.63	24.26 ± 4.90	**0.025**
Underweight	15 (28.8%)	4 (7.2%)	
Normal weight	26 (50%)	23 (41.8%)	
Overweight	5 (9.6%)	8 (14.54%)	
Obese	6 (11.53%)	20 (36.36%)	
**Smoking status** (*n*)			**0.001**
Current	8 (15.3%)	0	
Former	12 (23.07%)	6 (10.9%)	
Never	32 (61.53%)	49 (89.09%)	
**Alcohol status** (*n*)			0.054
Current	5 (0.6%)	0	
Former	9 (17.3%)	9 (16.36%)	
Never	37 (71.15%)	46 (83.63%)	

All the data is presented in mean ± standard deviation (SD) or number (*n*) and %.

*p-value was statistically significant at *p* ≤ 0.05, significant values are marked bold.

^ǂ^BMI, Body Mass Index.

### 3.2 Clinico-epidemiological data

**Figure 2:**
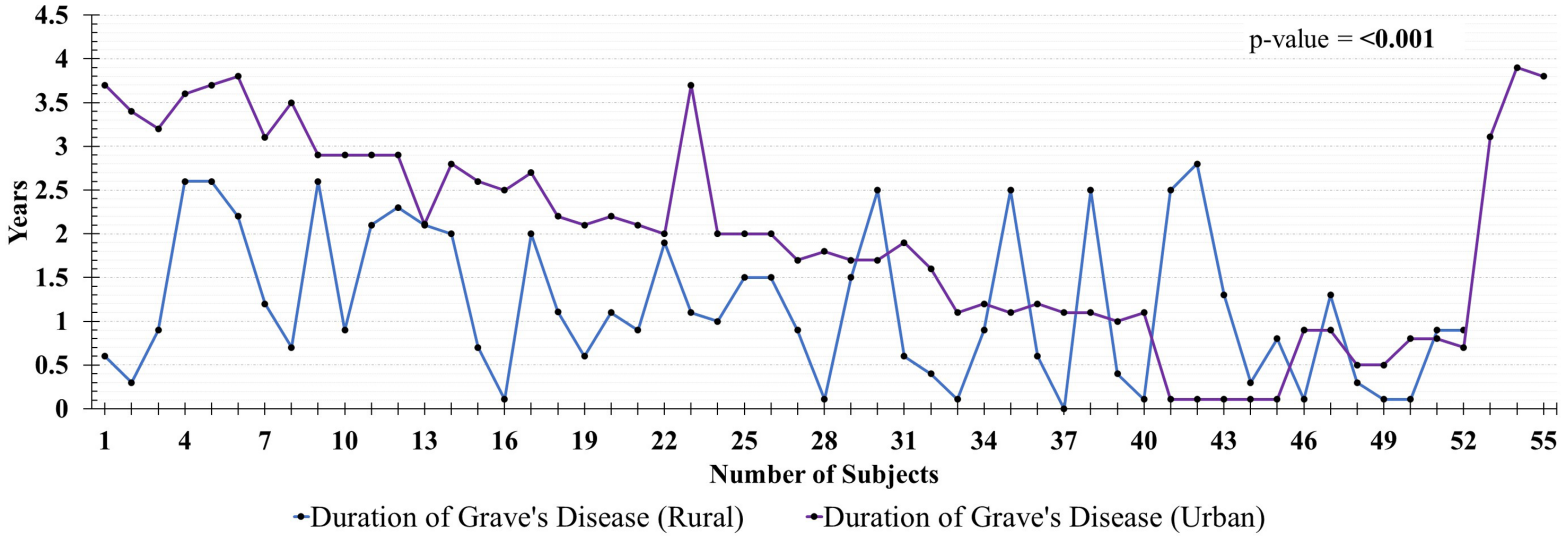
Comparison of duration of Grave’s disease between rural and urban patients.

The demographic parameters of the Graves patients presenting to rural and urban centers were compared and shown in Table 1. A significant difference was observed in female to male ratio in urban (3.1:1) and rural (1.2:1) settings (*p* value = 0.014). Moreover, patients from urban exhibited a higher level of education than those from rural centers, with respective counts of *n* = 48 and *n* = 7 (*p* value = <0.001). Urban patients have a longer duration of grave’s disease as compare to the rural patients (*p*-value = <0.001) (Figure 2).

**Figure 3:**
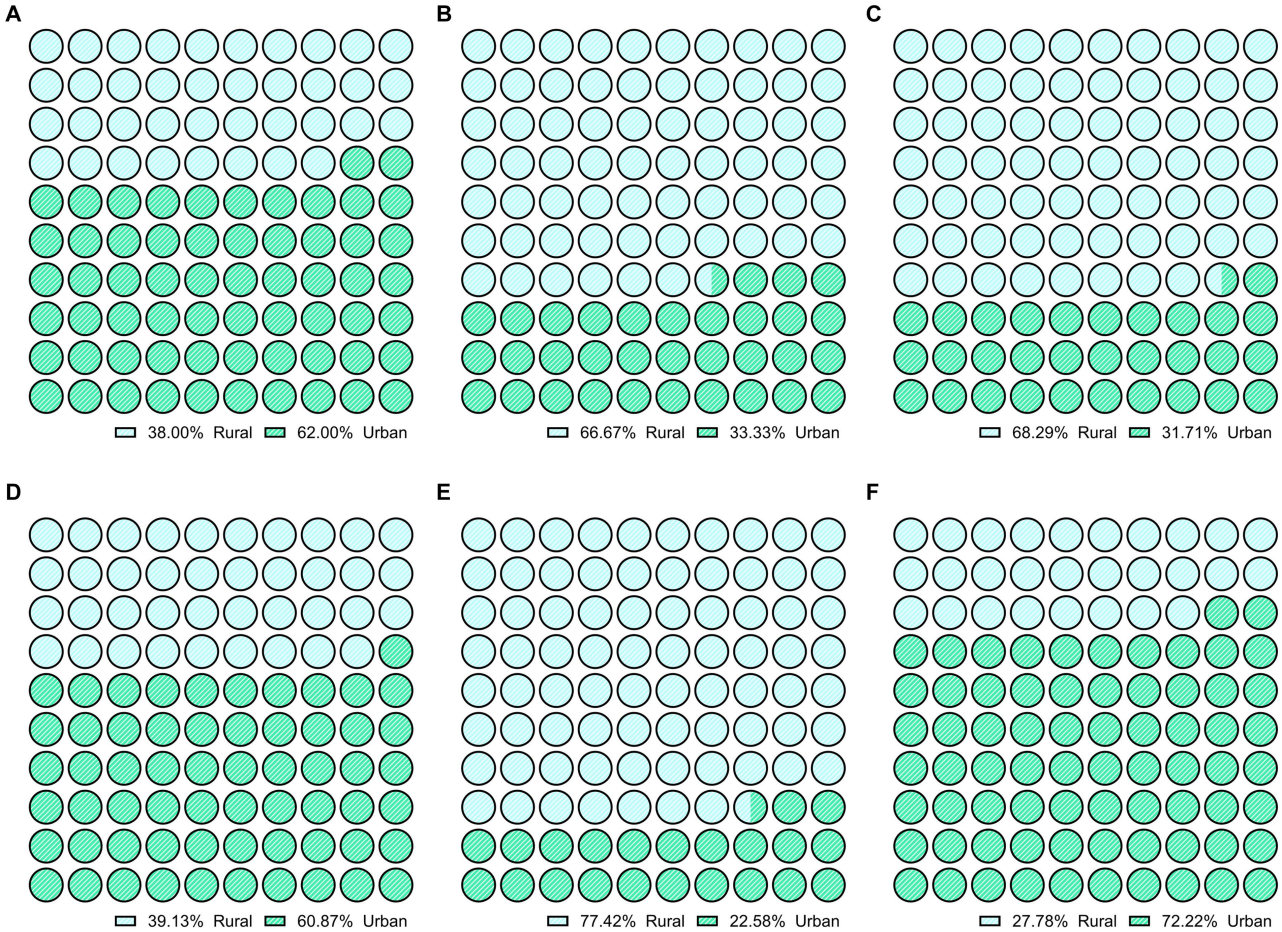
Comparison of clinical symptoms like **(A)** weight loss, **(B)** weight gain on therapy, **(C)** exophthalmos, **(D)** goiter, **(E)** nervousness, **(F)** tremor in rural and urban patients with Graves’ disease (*p*-value < 0.05).

A higher proportion of rural patients (23.07%) quit smoking than their urban counterparts (10.9%) (*p* value = 0.001). Patients from the rural center have lower body mass index than urban patients (20.94 ± 3.63 vs. 24.26 ± 4.90, kg/m^2^, *p*-0.025). Obesity was more prevalent in urban (36.36%, *n*-20) patients than in rural (11.53%, *n*-6) (*p* value = 0.002). After starting the treatment, any weight gain was more common in patients from rural centers (*n* = 16) than in urban centers (30.76% vs. 14.54%) (*p*-value = 0.022). Exophthalmos was more commonly observed in patients from rural centers (*n* = 28) than in those from urban centers (*n* = 13) (*p*-value = <0.001) as well as nervousness (*p*-value = <0.001). In contrast, goiter (*p*-value = 0.037) and tremor (*p*-value = <0.001) were more prevalent among urban patients compared to rural patients (Figure 3).

### 3.3 Lab investigations

Various laboratory diagnostic parameters were measured, including blood hemoglobin, serum thyroid stimulating hormone, serum total T3, and serum total T4. At presentation, serum, TSH levels were significantly higher in patients from urban centers compared to those from the rural centers (0.02 [0.01–0.72] mIU/L vs. 0.01 [0.00–0.02] mIU/L, *p*-value = 0.000). There were no significant differences in other diagnostic parameters between rural and urban center patients, including hemoglobin levels (12.45 [10.40–14.25] mg/dL vs. 12.70 [10.70–13.20] mg/dL, *p*-value = 0.880), T3 (triiodothyronine) levels ([1.77–8.68] ng/mL vs. 5.03 [3.19–7.84] ng/mL, *p*-value = 0.322), and T4 (thyroxine) levels (11.45 [7.82–14.02] mcg/dL vs. 12.17 [8.35–18.28] mcg/dL, *p*-value = 0.676) (Table 2).

**Table 2:** Clinical features of the Graves patients as per the urban and rural perspective.

Variables	Rural	Urban	*p*-value*
Lab parameters			
Hb^Ϯs^, median [Q1–Q2]	12.45 [10.40–14.25]	12.70 [10.70–13.20]	0.880
Male [12.3–16.4 mg/dL]	14.10 [11.80–15.10]	14.20 [12.65–15.03]	0.841
Female [11.0–14.4 mg/dL]	11.60 [9.55–12.80]	12.00 [10.55–13.20]	0.257
TSH^ϯ^, median [Q1–Q2], 0.5–5.0 mIU/L	0.01 [0.00–0.02]	0.02 [0.01–0.72]	**0.000**
T3^ϴ^, median [Q1–Q2], 0.9–2.8 ng/mL	3.86 [1.77–8.68]	5.03 [3.19–7.84]	0.322
T4^Ϫ^, median [Q1–Q2], 5.4–11.5 mcg/dL	11.45 [7.82–14.02]	12.17 [8.35–18.28]	0.676
Symptoms (*p*-value > 0.05)			
Puffy eyes (*n*)	16 (30.76%)	11 (20%)	0.102
Blurred vision (*n*)	16 (30.76%)	11 (20%)	0.102
DOE (*n*)	16 (30.76%)	13 (23.63%)	0.222
Palpitation (*n*)	20 (38.46%)	26 (47.27%)	0.160
Tiredness (*n*)	32 (61.53%)	27 (49.09%)	0.118
Increased appetite (*n*)	17 (32.69%)	24 (43.63%)	0.124
Decreased appetite (*n*)	9 (17.30%)	24 (43.63%)	0.001
Hands hot (*n*)	19 (36.53%)	24 (43.63%)	0.229
Hands moist (*n*)	19 (36.53%)	24 (43.63%)	0.229

The data is presented in median [Q1–Q2] or number (*n*) and %.

*p-value was statistically significant at *p* ≤ 0.05, significant values are marked bold.

^Ϯ^Hb, Hemoglobin; ^ϯ^Thyroid stimulating hormone; ^ϴ^Triiodothyronine; ^Ϫ^T4, Thyroxine.

### 3.4 Physical and mental component summary scores in patients from rural and urban centers

**Figure 4:**
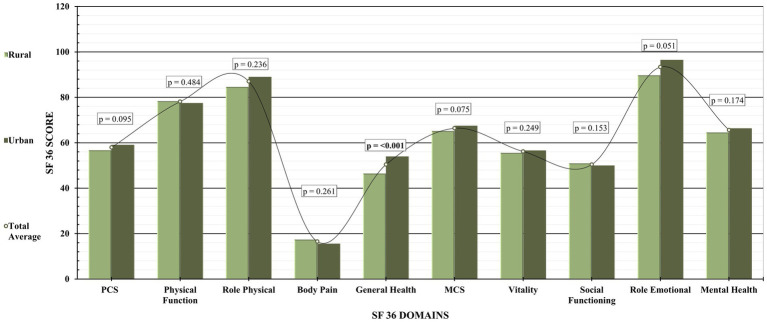
SF 36 multidomain scores of rural and urban patients.

The SF-36 Short Form health survey questionnaire was used to evaluate the scores of different domains. In the HRQOL questionnaire, urban patients had higher scores on general health (54.01 ± 8.03 vs. 46.44 ± 6.01, *p*-value = <0.001) and role emotional (96.49 ± 13.66 vs. 89.76 ± 26.00, *p*-value = 0.051) (Figure 4).

### 3.5 Association of baseline characteristics with physical component summary scores of SF-36

Multiple linear regression test was employed to investigate the relationship between various baseline characteristics and the physical component summary score. At rural centers, the duration of Graves’ disease negatively correlated with the physical component summary score [*β* = −0.435, −9.20 to 1.35, *p*-value = 0.010]. Also, an increase in the age of urban patients with Graves’ disease is linked to a decline in their physical component score [*β* = 0.38, −0.43 to −0.07, *p*-value = 0.005]. Moreover, urban patients’ Body Mass Index (BMI) is strongly associated with the physical component summary score [*β* = 0.30, −1.05 to −0.09, *p*-value = 0.021] (Table 3).

**Table 3:** Multivariable linear regression model factors associate with the physical component summary score of the SF-36 among urban and rural patients with Graves’ disease.

Factors	Rural			Urban		
	*β*	95% CI	*p*-value	*β*	95% CI	*p*-value
(Constant)		51.94–98.85	0.000		79.38–110.21	0.000
**Gender**						0.15
Female	−0.153	−10.72 to 4.46	0.411	−0.18	−9.53 to 1.54	
Male (ref)						
Age	0.10	−0.19 to 0.38	0.51	−0.38	−0.43 to −0.07	**0.005**
**Smoking**						
Current	0.004	−0.136 to 0.321	0.984	−0.16	−12.44 to 2.91	0.21
Former	0.130	−0.042 to 0.602	0.440	0.070	−1.79 to 3.46	0.664
Never (ref)						
BMI	−0.20	−1.36 to 0.21	0.148	−0.30	−1.05 to −0.09	**0.021**
Duration of Grave’s disease	−0.435	−9.20 to 1.35	**0.010**	−0.13	−3.04 to 0.82	0.25
Total symptoms	−0.324	−1.92 to 0.05	**0.039**	0.081	−0.56 to 1.15	0.49
	*R* = 0.47, *R*^2^ = 0.22, Adjusted *R*^2^ = 0.11 *F* = 2.07, *p* = 0.025, *β*_0_ = 75.39			*R* = 0.60, *R*^2^ = 0.36, Adjusted *R*^2^ = 0.29 *F* = 4.68, *p* = <0.001, *β*_0_ = 94.79		

*p-value was statistically significant at *p* ≤ 0.05, significant values are marked bold.

### 3.6 Association of baseline characteristics with mental component summary

Multiple linear regression was used to compare baseline attributes to the mental component summary score. Female patients with Graves’ disease in rural centers had higher mental component summary scores than male patients (*β* = 0.14, −1.70 to 2.29, *p*-value = 0.037). Rural patients with Graves’ disease had an inverse correlation between mental component score and disease duration (*β* = 1.076, −6.74 to 1.63, *p*-value = 0.026). In urban patients with Graves’ disease, age also had a negative correlation with mental component score (*β* = −0.662, −0.97 to 1.11, *p*-value = 0.009) (Table 4).

**Table 4:** Multivariable linear regression model factors associate with the mental component summary score of the SF-36 among urban and rural patients with Graves’ disease.

Factors	Rural			Urban		
	*β*	95% CI	*p*-value	*β*	95% CI	*p*-value
(Constant)		50.07–95.36	0.000		57.24–78.68	0.000
Gender	0.14	−1.70 to 2.29	**0.037**	−0.069	−5.56 to 3.58	0.665
Female						
Male (ref)						
Age	0.152	−0.08 to 0.52	0.157	−0.662	−0.97 to 1.11	**0.009**
Smoking						
Current	5.677	−11.10 to 11.79	0.952	0.033	−5.69 to 7.00	0.836
Former	4.261	−6.622 to 10.95	0.581	−0.053	−566 to 3.833	0.722
Never (ref)						
BMI	0.411	−1.30 to 0.355	0.256	0.001	−0.39 to 0.40	0.995
Duration of Grave’s disease	1.076	−6.74 to 1.63	**0.026**	0.076	−1.19 to 2.01	0.610
Total symptoms	0.502	−1.81 to 0.21	0.118	−0.573	−0.83 to 0.88	**0.019**
	*R* = 0.45, *R*^2^ = 0.22, Adjusted *R*^2^ = 0.21 *F* = 2.87, *p* = 0.025, *β*_0_ = 72.72			*R* = 0.44, *R*^2^ = 0.25, Adjusted *R*^2^ = 0.22 *F* = 2.18, *p* = 0.008, *β*_0_ = 67.96		

*p-value was statistically significant at *p* ≤ 0.05, significant values are marked bold.

## 4 Discussion

This is the first study of its kind, in which we conducted a comprehensive analysis comparing the clinical and epidemiological aspects of Graves’ disease in patients presenting at rural and urban centers, as well as investigating the factors influencing the quality of life among people with Graves’ disease. This study’s findings also revealed significant differences between rural and urban patients with Graves’ disease.

Compared to the urban endocrine center, patients with Graves’ disease who presented to the NIMS university’s rural endocrine center faced some unique challenges. They had a longer duration of disease, a lower BMI, more weight loss, and higher T4 levels at the time of presentation to the endocrine center. These indicate more severe disease in the patients who are presenting to the rural endocrine center of the NIMS university. Concurrently, their quality of life was lower than that of their urban counterparts.

Low awareness of Graves’ disease, poor rural health infrastructure, inability to diagnose Graves’ disease at the primary care level in India, low education status, poor nutrition, and more prevalent smoking in rural Graves patients were all factors contributing to this unique phenotype in rural Graves patients (23).

Urban patients were more educated and health-aware. However, rural patients often lack this awareness due to limited educational resources and healthcare infrastructure development. Awareness led to an early presentation at the urban endocrine center.

Underdeveloped healthcare facilities in rural India delayed Graves’ disease cases’ presentation to tertiary care centers that could treat it. This delay in seeking medical attention worsened disease symptoms, as rural patients had higher T4 levels. As mentioned, rural patients were more likely to smoke, which increased Graves’ disease risk and reduced the efficacy of the medical treatment. Smoking increases thyroid hormone production and decreases antithyroid treatment efficacy, which may explain rural patients’ higher T4 levels (23).

Poor HRQOL SF-36 scores indicated a low quality of life in rural Graves’ disease patients. The late presentation of rural patients to the university endocrine center prolonged untreated Graves’ disease. Long-term untreated Graves’ disease resulted in increased weight loss and sarcopenia, resulting in physical deconditioning (24). Deconditioning made daily activities more difficult and reduced physical component summary scores. In contrast, the Graves’ disease urban patient had better nutrition, lower T4 levels, earlier access to healthcare, and better follow-up, all of which improved physical health scores.

Rural Graves patients scored lower in mental health. Rural patients had lower mental health scores due to lower educational attainment, inadequate healthcare infrastructure, and elevated T4 levels, which caused mental frustration and lower mental health scores.

In literature, there is minimal data regarding the quality of life in the urban and rural Graves patients, making comparison difficult with the other studies.

Our study has some strengths and weaknesses. Understanding Graves’ disease from different geographical and socioeconomic settings makes our study unique. Graves patients’ quality of life was previously unexplored in literature, making our study the first of its kind. This study fills a need in medical research by comparing urban and rural quality of life and revealing how geographical and socioeconomic factors affect Graves’ disease patients’ health. This novel approach adds to existing knowledge and allows for more tailored and effective healthcare strategies for patients with this condition in diverse settings. The regional nature of our sample may limit the generalizability of our findings to the broader population of patients with this condition, in addition to potential concerns about sample size. The rarity of Graves’ disease could introduce selection bias. Further research using larger and more diverse cohorts is needed to validate our results. There is no health control group to compare the Graves’ HRQOL to that of a healthy population. It would also be interesting to follow up on these patients to see how effective the medical treatment is.

## 5 Conclusion

In conclusion, this study highlights significant disparities in the clinical and quality of life aspects of Graves’ disease between rural and urban patients in India. The findings underscore the urgent need for improved healthcare infrastructure and awareness programs in rural centers, as well as the importance of addressing smoking as a potential risk factor.

## Data Availability

The raw data supporting the conclusions of this article can be made available by a suitable request to the corresponding author(s).

## References

[1] Weetman AP. Graves’ disease. N Engl J Med. (2000) 343:1236–48. doi: 10.1056/NEJM200010263431707, PMID: 11071676

[2] Jin J Sandoval V Lawless ME Sehgal AR McHenry CR. Disparity in managing Graves’ disease observed at an urban county hospital: a decade-long experience. Am J Surg. (2012) 204:199–202. doi: 10.1016/j.amjsurg.2011.10.010, PMID: 22317948 PMC3623456

[3] McLeod DS Cooper DS Ladenson PW Whiteman DC Jordan SJ. Race/ethnicity and the prevalence of thyrotoxicosis in young Americans. Thyroid. (2015) 25:621–8. doi: 10.1089/thy.2014.0504, PMID: 25744381

[4] Wanjari M Patil M Late S Umate R. Prevalence of thyroid disorder among young adults in the rural Centre of Wardha district: a cross-sectional study. J Family Med Prim Care. (2022) 11:7700. doi: 10.4103/jfmpc.jfmpc_806_22, PMID: 36994012 PMC10041002

[5] Rajasthan District List, Jaipur District, Jaipur Population, Population Census 2011, Office of the Registrar General & Census Commissioner, India. Ministry of Home Affairs, Government of India. Available at: https://censusindia.gov.in/census.website/ (Accessed March 8, 2024).

[6] Mudey A Ambekar S Goyal RC Agarekar S Wagh VV. Assessment of quality of life among rural and urban elderly population of Wardha District, Maharashtra, India. Stud Ethno-Med. (2011) 5:89–93. doi: 10.1080/09735070.2011.11886394

[7] Kahaly GJ Bartalena L Hegedüs L Leenhardt L Poppe K Pearce SH. 2018 European thyroid association guideline for the management of graves’ hyperthyroidism. Eur Thyroid J. (2018) 7:167–86. doi: 10.1159/000490384, PMID: 30283735 PMC6140607

[8] Van Uytfanghe K Ehrenkranz J Halsall D Hoff K Loh TP Spencer CA. ATA thyroid function tests writing group. Thyroid stimulating hormone and thyroid hormones (triiodothyronine and thyroxine): an American Thyroid Association-commissioned review of current clinical and laboratory status. Thyroid. (2013) 33:1013–28. 10.1089/thy.2023.0169 PMC10517335 37655789

[9] Tham KW Abdul Ghani R Cua SC Deerochanawong C Fojas M Hocking S. Obesity in south and Southeast Asia—a new consensus on care and management. Obes Rev. (2023) 24:e13520. doi: 10.1111/obr.13520, PMID: 36453081 PMC10078503

[10] General Concepts, Glossary, National Health Interview Survey, National Center for Health Statistics, Centers for Disease Control and Prevention Available at: https://www.cdc.gov/nchs/nhis/tobacco/tobacco_glossary.htm#:~:text=Current%20smoker%3A%20An%20adult%20who,and%20who%20currently%20smokes%20cigarettes (Accessed March 10, 2023).

[11] Binge drinking, Glossary - Alcohol, National Health Interview Survey, National Center for Health Statistics, Centers for Disease Control and Prevention. Available at: https://www.cdc.gov/nchs/nhis/alcohol/alcohol_glossary.htm (Accessed March 10, 2023).

[12] Brazier JE Harper R Jones NM O'cathain A Thomas KJ Usherwood T. Validating the SF-36 health survey questionnaire: new outcome measure for primary care. Br Med J. (1992) 305:160–4. doi: 10.1136/bmj.305.6846.160, PMID: 1285753 PMC1883187

[13] Sinha R van den Heuvel WJ Arokiasamy P. Validity and reliability of MOS short form health survey (SF-36) for use in India. Indian J Commun Med. (2013) 38:22. doi: 10.4103/0970-0218.106623, PMID: 23559699 PMC3612292

[14] RAND, RAND Health Care, Surveys RAND Medical Outcomes Study, 36-Item Short Form Survey (SF-36). Available at: https://www.rand.org/health-care/surveys_tools/mos/36-item-short-form/scoring.html (Accessed March 06, 2023).

[15] Ware JE Jr Sherbourne CD. The MOS 36-item short-form health survey (SF-36): I. Conceptual framework and item selection. Med Care. (1992) 30:473–83. doi: 10.1097/00005650-199206000-00002, PMID: 1593914

[16] Hays RD Shapiro MF. An overview of generic health-related quality of life measures for HIV research. Qual Life Res. (1992) 1:91–7. doi: 10.1007/BF00439716, PMID: 1301123

[17] Steward AL Sherbourne C Hayes RD Wells KB Nelson EC Kamberg CJ. Summary and discussion of MOS measures In: Stewart AL Ware JE, editors. Measuring functioning and well-being: the medical outcome study approach. Durham, NC: Duke University Press (1992). 345–71.

[18] Marks JR Schectman JM Groninger H Plews-Ogan ML. The association of health literacy and socio-demographic factors with medication knowledge. Patient Educ Couns. (2010) 78:372–6. doi: 10.1016/j.pec.2009.06.017, PMID: 19773144

[19] You AS Kalantar-Zadeh K Brent GA Narasaki Y Daza A Sim JJ. Impact of thyroid status on incident kidney dysfunction and chronic kidney disease progression in a nationally representative cohort. Mayo Clin Proc. (2024) 99:39–56. doi: 10.1016/j.mayocp.2023.08.028, PMID: 38176833 PMC10795379

[20] Mohamed HN Ghedi AK Ozturk S Jeele MO Bashir AM. Hypokalemic periodic paralysis as the first sign of thyrotoxicosis-a rare case report from Somalia. Thyroid Res. (2023) 16:14. doi: 10.1186/s13044-023-00158-4 37303055 PMC10258971

[21] Lee J Yi S Kang YE Kim HW Joung KH Sul HJ. Morphological and functional changes in the thyroid follicles of the aged murine and humans. J Pathol Transl Med. (2016) 50:426. doi: 10.4132/jptm.2016.07.19, PMID: 27737529 PMC5122729

[22] Santos TD Oliveira JC Freitas C de Carvalho AC. Thyroid-stimulatory antibody as a predictive factor for graves’ disease relapse. Cureus. (2022) 14:e22190. doi: 10.7759/cureus.22190, PMID: 35178331 PMC8843073

[23] Eckstein A Quadbeck B Mueller G Rettenmeier AW Hoermann R Mann K. Impact of smoking on the response to treatment of thyroid associated ophthalmopathy. Br J Ophthalmol. (2003) 87:773–6. doi: 10.1136/bjo.87.6.773, PMID: 12770979 PMC1771717

[24] Elberling TV Rasmussen AK Feldt-Rasmussen U Hording M Perrild H Waldemar G. Impaired health-related quality of life in Graves’ disease. A prospective study. Eur J Endocrinol. (2004) 151:549–55. doi: 10.1530/eje.0.1510549, PMID: 15538931

