# Supplementary material for: A comparative cross-sectional study on the quality of life in Grave’s disease patients: urban vs. rural perspectives
Source: Front Public Health. 2024 Aug 21;12:1345803. doi: 10.3389/fpubh.2024.1345803 (PMC11371591; doi:10.3389/fpubh.2024.1345803)
Supplement: Supplementary file 1 [file Data_Sheet_1.DOCX]

# Sample Size Calculation for QoL Study in Graves' Disease Patients

To calculate the sample size for comparing the quality of life (QoL) in patients with Graves' disease in rural and urban areas using the SF-36 questionnaire, we use the formula for comparing two means. Although there is no specific study on this topic, we extrapolate from past studies comparing QoL in rural and urban populations without Graves' disease.

## Formula for Sample Size Calculation

n = 2 * sigma^2 * (Z_alpha/2 + Z_beta)^2 / d^2

Where:
n = sample size for each group (rural and urban)
sigma = standard deviation of the QoL scores
Z_alpha/2 = Z value for the desired confidence level (95%), which is 1.96
Z_beta = Z value for the desired power (80%), which is 0.84
d = the effect size (difference in means between the two groups)

## Assumptions

Let's assume:
1. The standard deviation (sigma) of the SF-36 scores is 10 (this value can be adjusted based on previous studies or pilot data).
2. The expected difference in the mean SF-36 scores between rural and urban areas (d) is 11.

To recalculate the sample size for the QoL study in Graves' disease patients with a standard deviation (sigma) of 10 instead of 20, we use the same formula:
sigma = 10
Z_alpha/2 = 1.96 (for 95% confidence level)
Z_beta = 0.84 (for 80% power)
d = 11 (expected difference in mean SF-36 scores between rural and urban areas)
Putting in these values into the formula:
n = 2 * 10^2 * (1.96 + 0.84)^2 / 11^2
First, calculate (1.96 + 0.84)^2:
(1.96 + 0.84) = 2.8
(2.8)^2 = 7.84
Then calculate the numerator:
2 * 10^2 * 7.84 = 2 * 100 * 7.84 = 1568
Now, calculate the denominator:
11^2 = 121
Finally, divide the numerator by the denominator:
n = 1568 / 121 ≈ 12.96
Therefore, you will need approximately 13 participants in each group (rural and urban) to achieve 95% confidence and 80% power for detecting a difference of 11 in the QoL scores measured by SF-36, assuming a standard deviation of 10. OpenEpi has showed the same sample size.

We have reviewed our EMR database and took eligible patients from both urban and rural centers.
